# Abiotic and Biotic Factors Regulating Inter-Kingdom Engagement between Insects and Microbe Activity on Vertebrate Remains

**DOI:** 10.3390/insects8020054

**Published:** 2017-05-24

**Authors:** Heather R. Jordan, Jeffery K. Tomberlin

**Affiliations:** 1 Department of Biological Sciences, Mississippi State University, Starkville, MS 39705, USA; 2 Department of Entomology, Texas A&M University, College Station, TX 77843, USA

**Keywords:** decomposition, insect, microbe, microbiome, abiotic, biotic, inter-kingdom, colonization

## Abstract

A number of abiotic and biotic factors are known to regulate arthropod attraction, colonization, and utilization of decomposing vertebrate remains. Such information is critical when assessing arthropod evidence associated with said remains in terms of forensic relevance. Interactions are not limited to just between the resource and arthropods. There is another biotic factor that has been historically overlooked; however, with the advent of high-throughput sequencing, and other molecular techniques, the curtain has been pulled back to reveal a microscopic world that is playing a major role with regards to carrion decomposition patterns in association with arthropods. The objective of this publication is to review many of these factors and draw attention to their impact on microbial, specifically bacteria, activity associated with these remains as it is our contention that microbes serve as a primary mechanism regulating associated arthropod behavior.

## 1. Introduction

Forensic entomology is the well-established field of applying insect science to aid legal investigations where arthropods are associated with living [1,2], or deceased, people [3,4], pets [5], wildlife [6], or even livestock [7]. Historically, forensic entomologists have been asked to determine the time of death (i.e., postmortem interval (PMI)) [8,9] in cases involving decomposing remains. Recently, however, this activity has been called into question [10,11] with entomologists instead determining the age of insects collected from victims, in order to estimate a time of colonization, which could differ from the actual time of death (e.g., before death due to myiasis or after death resulting in a minimum PMI).

Regardless, both analyses are built upon a foundation of assumptions regarding insect activity that might not always be completely accurate, and could, therefore, impact the validity of downstream information and inferences. For instance, presuppositions, such as assuming colonization occurred after death [1,12], insect material collected originally from the remains in question [13], the development of datasets from one region are applicable to insect populations from other regions [14,15,16], or even abiotic conditions [7,17,18,19,20,21,22,23] at the time of death may have influenced insect activity and subsequent colonization, are important considerations in determining the time of colonization. However, presupposed estimates of such insect attributes may not necessarily line up with the actual time of death or colonization, respectively, under all circumstances [13]. 

Many factors impacting insect activity in association with decomposing human remains have been recognized previously, where differences in estimates versus actual insect colonization, development, and associated succession were coupled with factors impacting the insect(s) directly. While conditions of temperature, light, access to the remains, and precipitation do, indeed, impact the behavior of forensically-important insects [7], other determinants, such as microbial colonization and physiology associated with the remains or with the insects, are now recognized as important influences on the behavior of forensically-relevant arthropods [24,25,26,27]. However, little is known of specific microbial-insect inter-kingdom engagement and the resulting impact on insect activity. More specifically, how do microbes, whether associated initially with the insect or the remains, play a role in regulating arthropod detection, attraction, and colonization of decomposing remains? 

While research in this arena is still in its infancy, early data indicate bacteria associated with decomposing remains could be a major factor regulating these behaviors. In fact, those abiotic factors previously listed that are known to impact insect behavior are also factors regulating microbial activity. Therefore, such abiotic influences to bacteria likely result in a perturbation through the system impacting higher trophic levels that are leading to the often-observed shifts in arthropod behavior. However, before diving into these factors and how they might facilitate the behaviors observed by arthropods associated with decomposing remains, an understanding of ecological perspective of natural processes associated with nutrient recycling of such resources is in order.

Vertebrate remains, like all decomposing material, for the most part, represent limited resources in nature [28]. As pointed out numerously in other publications, these remains are, in most cases, unpredictable in nature, and rich in nutrients; thus, making them attractive locations for colonization, reproduction, and food sources. Consequently, animals that compete for these resources are under intense pressure to quickly locate and consume them to avoid starvation, competition [29], or failure to locate a mate [30,31].

### 1.1. A Need for Integrative Research of Entomology and Microbiology 

Limited research exploring insect-microbe interactions as related to ecological aspects of carrion degradation has been conducted [28]. Furthermore, entomological research of carrion ecology historically focused attention on post-colonization [32,33,34,35,36]. However, two recent publications have drawn attention to the pre-colonization interval [37], or pre-appearance interval [38], as being important to understand as well. These studies indicate that factors, such as temperature, and carrion and microbe-derived odors, drive attraction and subsequent colonization, and are critical to account for the elapsed time from death to discovery [37]. 

Until recently, microbes associated with decomposing material were often thought of simply as facilitators or recyclers of nutrients; however, Janzen [39], in his landmark publication, discussed the various roles of microbes beyond a simple nutrient recycler. In fact, recent data demonstrate Janzen was correct in his assertion that microbes are much more—they fall into most ecological categories including, but not limited to, competitor [40,41], mutualist [42,43], or even predator [44]. 

This new paradigm gives a completely different spin to appreciating insect activity associated with decomposing remains; roles bacteria and other microbes play with regards to their ecological and forensic relevance can be applied within the context of factors driving insect attraction, colonization, and utilization. In this same context, insect activity may also have an equal or opposite effect on microbial colonization and function during decomposition processes. However, it should be noted that outside of a few laboratory studies, which will be discussed later, very little is known about the complete microbial community associated with decomposing remains, associated with the arthropod, or the microbial interactions between the two. 

### 1.2. What Is Known about Microbes Associated with Decomposing Vertebrate Remains?

**Figure 1:**
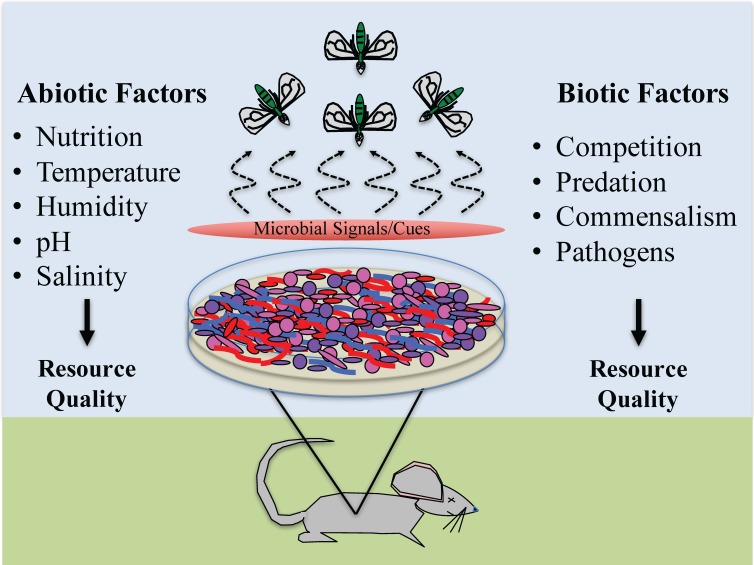
Factors impacting microbial activity that may also impact arthropod activity associated with decomposing remains. Figure designed by Jeffery K. Tomberlin and Heather R. Jordan.

Researchers are still describing microbial communities associated with decomposing remains and, in most cases, descriptions and cataloging are restricted to bacteria [45,46,47,48,49]. Who is present and how these communities differ across resources are still not fully known, which presents wonderful opportunities for exploratory research and applications in insect behavior and ecology as related to forensics. Many of the currently published forensically-relevant studies of microbial communities are predominately hypothesis driven, examining the impact of abiotic and biotic factors potentially influencing microbial composition. The purpose of this paper is to present a different perspective of the decomposition process and provide some basic understanding of some, but certainly not all, factors impacting microbial activity which, in turn, likely impacts arthropod behavior (Figure 1). Though many of these factors are known, especially to microbiologists, we hope that this paper will provide concepts of the regulation of microbe-insect interactions, in order to further guide considerations of such forensically-relevant evidence.

## 2. Abiotic Factors

### 2.1. Temperature

As with arthropods, bacterial growth and activity are influenced by ambient temperature. Microorganisms can inhabit vastly different temperature ranges, which contain a minimum, maximum, and optimal temperature for growth. Temperature is one of the most important parameters regulating the activities of microorganisms. Due to the impact of temperature on all reactions of the cell, such as changes in membrane fluidity, cellular pH, and protein integrity, to name a few (Table 1), adaptation to fluctuations in temperature is possibly the most common response researched [50,51,52,53].

The intrinsic metabolic rate, and consequently the energy demand, may be inflated with increasing temperature [51,60]. Therefore, increases in temperature with only limited nutrient availability may have a negative effect on growth due to the fact that metabolism increases, though the energy needed for these processes is not available. If, instead, food resources are well-supplied, the organisms have a surplus of energy, which can be invested in biomass growth. Under these conditions, microbial biomass would increase with temperature up to a certain point where enzymes and other cellular processes cease to function. Hence, the microbial growth response becomes more complex if resource availability varies in concert with temperature. 

Sensitivity of cells to cold stress is dependent on several factors including temperature, rate of cooling/freezing, culture medium, microbial strain, and duration of storage [61]. Incubation at low temperatures can also change the lipid composition of microbial cells. Many microorganisms, including some bacteria and yeasts, contain an increasing proportion of unsaturated fatty acids as the growth temperature decreases [62,63]. This increase in the proportion of unsaturated fatty acids with decreasing temperature is believed to be essential for membrane function at low temperatures. When the temperature is lowered, some of the normally fluid components become gel-like, which prevents proteins from functioning correctly, resulting in bacterial membrane leakage. Therefore, the change in unsaturated fatty acids allows retention of membrane fluidity as temperature decreases, preventing viscosity and allowing the bacteria to continue to grow.

Shifts in environmental conditions away from the optimal not only cause changes in microbial growth, but also lead to the induction of many stress responses (i.e., modulation of genes in response to heat or cold shock, starvation, or desiccation). These strategies are generally directed at survival rather than growth. For instance, an abrupt change in temperature may lead to gene expression as a global stress response to either cold or heat where shock proteins are induced under these conditions in an attempt to prevent cell death [64,65]. 

Therefore, in the context of carrion decomposition, associated microorganisms are heavily influenced by the varying internal and external temperatures of the cadaver, which are heavily guided by the ambient temperature. Insect colonization also impacts cadaver temperature, as maggot activity generates a large amount of heat [66,67]. These temperature responses can be seen manifested as changes in carrion decomposition rates. Several studies have reported larger nutrient-induced increases in microbial biomass and enzymatic activity in warmer temperature than in cooler temperatures [68,69], and a slower rate of decomposition in winter months [70]. Volatile organic compounds (VOCs) associated with decomposition were also detected in higher abundance in summer months than in winter months, corresponding also to increased insect activity [70]. However, other studies have shown that microbially-mediated decomposition is most efficient at lower temperatures, and that microbial functional activity, as measured using Biolog Ecoplates, increased across winter months [71,72]. The authors speculate this more efficient decomposition may be due to lower microbial catabolic requirements at lower temperature and perhaps a lack of competition with invertebrates or vertebrate scavengers [71]. Discrepancies in results between studies and temperatures may reflect variance in study design and underscore the importance of further studies using standardized study methodology to understand seasonality associated with decomposition [73].

### 2.2. Water Activity

Microorganisms, like all living organisms, require available water to grow and function. Different types of fungi or bacteria require varying amounts of water to reproduce and grow, and the production of enzymes and other metabolites may be sensitive to alterations. The majority of microorganisms require 60% relative humidity (RH) or more, though some can survive and multiply in >20% RH [74]. Thus, decreasing temperature and moisture (relative humidity), creates a less hospitable environment for microorganisms to grow. One indicator of microbial response is their taxonomic classification. For example, Gram-negative bacteria are generally more sensitive to low water activities (aw, partial vapor pressure of water in a substance divided by the standard state partial vapor pressure of water) than Gram-positive bacteria [74]. Many bacterial pathogens are controlled at aw well above 0.86, whereas others, such as *Staphylococcus aureus*, can grow and produce toxin at aw as low as 0.86 [74,75]. Additionally, at high aw, fungi compete with bacteria, where pH then becomes a key driver of community assembly [76]. The best evidence that microbial taxa differ in moisture-related niches comes from culture-based studies of soil microbial communities where one study showed microbial taxa differed in their ability to enter into dormant states, and also to quickly respond to optimal conditions [77]. Though it is unclear whether these observations reflect field conditions and/or interactions with other taxa, these functional traits are likely to affect survival under rapid changes in soil water potential and allow prediction of microbial structure and functioning under differing moisture levels in many systems [77].

Insects are also affected by their water content, and humidity influences their survival and resistance to desiccation [78]. Sensing these conditions is crucial to arthropods as this helps to estimate the danger of water loss when emerging from cover, which could, in turn, impact resource colonization. Insect hygroreception has been well documented in many species [79]. Mechanisms by which atmospheric humidity stimulates hygroreceptive cells remains a mystery in many insect systems, though several models have been developed [80]. A study by Tichy [81] evaluated the humidity transduction models in *Periplaneta Americana* (Linnaeus) (Blattodea: Blattidae), and found that responses of the cockroach’s moist and dry cells to slow and continuous changes in vapor pressure depend on temperature, and that the psychrometer model in which the cooling effect of water evaporation is used to measure humidity was the best fit [80]. Additionally, differences in water content, and also humidity, impact insect responses to chemical signals, which may differ in their water vapor release [81]. 

**Figure 2:**
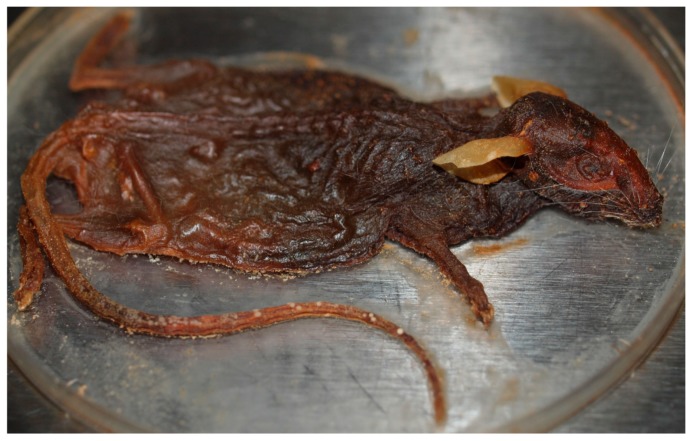
Mummification brought about through warm, dry conditions (photograph courtesy of Heather R. Jordan).

These parameters may also be applied to both insect and microbial colonization of vertebrate remains, as the cadaver landscape can be considered as representing multiple microenvironments. The natural preservation of a cadaver is highly dependent on the surrounding environment, and a range of factors can play a part in this phenomenon, including temperature, humidity, and the action of bacteria and other microorganisms. Microbial taxa shift their responses as their local landscape changes. Shifts in the microbial community composition under altered moisture conditions suggest differential sensitivity to moisture, but total DNA abundance within each taxon could reflect long-term legacies preceding moisture manipulation. Microbial VOC emissions would presumably be influenced by water vapor release rates of the cadaver sources that could alter insect colonization behavior. In this case, insect colonization of vertebrate remains could be dictated by the humidity of the remains, by the presence or absence of airborne chemicals arising from the remains and/or microbes, or a combination of both. To illustrate this, cadavers placed at higher humidity and at lower temperatures took longer to decompose and stayed wet longer [48]. Furthermore, cold and dry temperatures significantly slow microorganism and insect activity, reducing the rate of decomposition and bringing about mummification [73,82,83] (Figure 2). 

Hot, dry climates can also hinder bacterial growth, limiting the bacterial decay and further preserving the body. Under such conditions, moisture can evaporate from the skin at such an accelerated rate that the process of mummification can occur. The internal organs may be preserved to an extent, though they will typically undergo some level of decomposition, and so are likely to be smaller in size. 

### 2.3. Resource Quality

Microorganisms play an essential role in nutrient recycling and those organisms that help break down dead plants and animals play a role in the recycling of carbon in the environment. Bacteria, and many fungi, perform chemical reactions on the tissues of decaying organic matter and convert carbon-containing compounds into carbon dioxide and water, or to methane by bacteria, depending on aerobic or anaerobic conditions. Results from microbially-mediated decomposition, resulting microbial biodiversity, and recycling and conversion of nutrients also influences insect attraction and resource acquisition, depending on the resulting quality and nutritional balance of the resource. Additionally, some microbes, such as some fungi, produce toxic compounds during colonization and decomposition, which may lead to insect avoidance of the resource [84], whereas other fungal species may produce fungal odors that are attractive semiochemicals to insects [85].

Most studies of nutrient recycling by microbes historically involved soil microbial communities and plant litter as detritus, with very few studies focused on carrion decomposition. However, recent attention to forensic microbiology, and the advent of next generation sequencing has allowed great advances and an appreciation for the role of microbes in carrion decomposition, and the utility in forensics. 

Resource-mediated changes in microbial activity have the potential to alter ecosystem processes, such as the production and respiration of organic material that will also impact decomposition. Changes in the temporal variability and quality of resource supply have been shown to influence enzyme activity, community composition, stoichiometry, and metabolic processes of heterotrophic bacterial and fungal communities in a variety of ecosystems [86,87,88]. For instance, as already stated, microbes release VOCs that are well-known attractants for insects in locating and determining the quality of a decomposing resource [89,90]. Indeed many arthropods respond to VOC stimuli associated with differing microbially-mediated stages of decomposition and at differing concentrations [91]. 

However, less is known about how microorganisms respond to resource quality variability, although their ability to adapt to rapid changes in their environment suggests dynamic changes in order to survive and compete with other organisms. Physiochemical conditions in a microenvironment are subject to rapid change in both time and space. During such events, certain populations may maintain a constant level of metabolic activity without showing a marked increase in numbers in response to the addition of an available nutrient source. Other organisms may have bursts of activity in response to the addition of available carbon and other nutrient sources, and may temporarily dominate the activities in the resource and grow to high numbers [86,92]. Understanding a microbial community’s nutrient requirements is key to the study of decomposition.

Almost immediately following host death, microbially-mediated decomposition begins, and commensal microbes begin to translocate following the cessation of host immune function, facilitated by the upregulation of genes for motility and production of exoenzymes for utility in breaking down tissues for nutrient availability [93]. Depending upon the placement of the cadaver, non-indigenous microorganisms, particularly soil microbial communities, may also “invade” the cadaver. As the local environment moves from aerobic to anaerobic, proliferation of anaerobic commensal microbes takes place, leading to the process of putrefaction, where carbohydrates, lipids, and proteins are bound and fermented, releasing gases [93,94,95]. In this same sense, insect colonization will also impact resource quality for the microbe. As insect colonization increases, so does the change in the local environment temperature, pH, and the input of insect-associated microbes. Additionally, insect colonization may make available new resources through internalizing into the cadaver, while also potentially changing the nutrient composition of the cadaver resource and oxygen conditions. 

The chemical composition of organic matter (i.e., cadaver resource), in turn affects, the microbial activity and community structure and, thus, the decomposition rate [96]. Carter et al. [68] reviewed several decomposition studies and showed that cadaver decomposition patterns differ from patterns seen from the microbial breakdown of plant and fecal matter, and that cadavers might not persist in terrestrial ecosystems as long as fecal matter and woody material [68]. The authors suggested the discrepancy between the pattern of cadaver and plant/fecal decomposition is probably due to the complexity of the substrate and presence of skin, which will retain cadaveric moisture, and the rate at which fly larvae assimilate cadaveric material [68]. In another study, mammal carcasses were found to have a narrow carbon to nitrogen ratio (5:1–8:1), high water content, and a wider variety of nutrients and faster decomposition rates than plant litter [97]. From an entomological perspective, nutrient composition of resources impacts the development of forensically-relevant arthropods [98]. However, how insects and microbes on these resources interact remains to be determined.

Within soil microbial communities, findings positively correlate substrate quality (particularly nitrogen content) and decomposition rates [97]. In these microbial communities, fungal biomass often contains more carbon (C) per unit nutrient than bacterial biomass with carbon:nitrogen (C:N) ratios of fungal ratios range from 10:1 to 15:1 and bacterial C:N ratios ranging from 3:1 to 12:1. Regardless of the proportion of bacteria and fungi in the community, overall microbial biomass has a ratio of 4:1 to 8:1. Studies have shown that low-quality resources (high C:N) favor fungi and high-quality (low C:N) resources favor bacteria [99,100]. These general ratios suggest that no matter the ecosystem and associated microbial community’s decomposition capacity, a set of common metabolic pathways stabilizes carbon in the soil long term, allowing the prediction of decomposition rates. Additionally, commonly-observed shifts in fungal:bacterial ratios during decomposition may be a result of shifts in resource C:N ratios that result in changes in microbial carbon-use-efficiency [101,102]. However, other factors such as nutrient solubility/availability [100], microbial community composition and/or resource history of that community may also be considered to contribute to the rate [103]. Consequently, there is a need for sensitive and systematic methods that can determine, or at least provide good indications of, the rates of production of bacteria and fungi during the decomposition process.

A large nutrient pulse, such as with the remains of a dead animal, may be followed by a period of nutrient deprivation. Due to this, microorganisms often face a feast or famine lifestyle. To offset this, microorganisms in some instances have storage reserves to draw upon. Bacteria may respond to variability in resource quality through a variety of mechanisms, including changes in the number of rRNA operons [104], extracellular enzyme activity, shifts in cellular morphology, entry into a viable, but non-culturable state [105], and storage of carbon or lipid reserves [106,107].

### 2.4. Narcotics

It is well known in forensic entomotoxicology that insect larvae can accumulate drugs, toxins, and poisons ingested by the deceased person, which can, in turn, delay insect colonization and/or development [108,109,110]. However, the role of chemical treatments—including medication and lifestyle interventions—that may confound the deceased individual’s microbial composition remain largely underexplored. 

Nevertheless, a few studies have investigated this question in the living host. For instance, in a mouse model, researchers determined the effect of phencyclidine (PCP) on recognition and impairment of mice, as well as associations between changes in the gut microbiome and those specific behavioral parameters [111]. Additionally, antibiotic- and control-treated rats were tested in the same manner. The researchers determined PCP treatment changed the microbiome, which was correlated with changes in mice behavior; however, it could be restored following PCP cessation [111]. Additionally, administration of ampicillin abolished the PCP-induced memory deficit, underscoring the microbe-narcotic-behavior relationship. 

In humans, a population-based metagenomics study found markers for gut microbiome diversity and composition [112]. Along with other metrics, such as smoking status, diet, and health state, antibiotic use was significantly associated with microbiome composition, particularly showing a decrease in two species from the genus *Bifidobacterium*, in line with other studies [113]. Several other drug categories, such as proton-pump inhibitors (PPI), statins, and laxatives, also had a strong effect on the gut microbiome, and PPI users were found to have profound changes in 33 bacterial pathways [112]. Furthermore, the authors found that metformin, a drug used to control blood sugar for type 2 diabetics, led to an increased abundance in *Escherichia coli* and a positive correlation with specific pathways, including the degradation and utilization of d-glucarate, d-galactarate, and pyruvate fermentation pathways. 

These data show that, in a living individual, drug use has a profound effect on the host microbiome diversity and richness, with the possibility of restoration to the original state following drug cessation. This has obvious implications for insect colonization and development. Not only do insects show a developmental response to active drug compounds [114], insects respond to microbial signaling compounds [26,115], and it is not known whether microbial signaling compound concentrations may be altered or shifted depending upon the deceased individual’s microbiome depending upon the drug class, time, and duration of ingestion. Thus, further research into drug-microbiome interactions and underlying mechanisms, as well as insect responses to such conditions, is needed.

## 3. Biotic Factors

### 3.1. Other Microbes

Microbial interactions on vertebrate remains could dictate which insects are attracted to, and colonize, them. Furthermore, insect colonization is also likely to dictate individual and microbial community response. The habitat a microbe resides in is governed by the physical and chemical conditions determined, in part, by the metabolic activities of other microbes within the community. For example, the organic material used by one species may have been a by-product of a second species. For any species to survive, they must exploit a novel niche or utilize resources more efficiently than the current dominant population. Some microbes may be involved in syntrophy, in which the metabolic activities of the organisms are mutually dependent, and only in combination are they thermodynamically favorable. In this instance, interacting microbes have adapted to a cooperative lifestyle [116].

**Figure 3:**
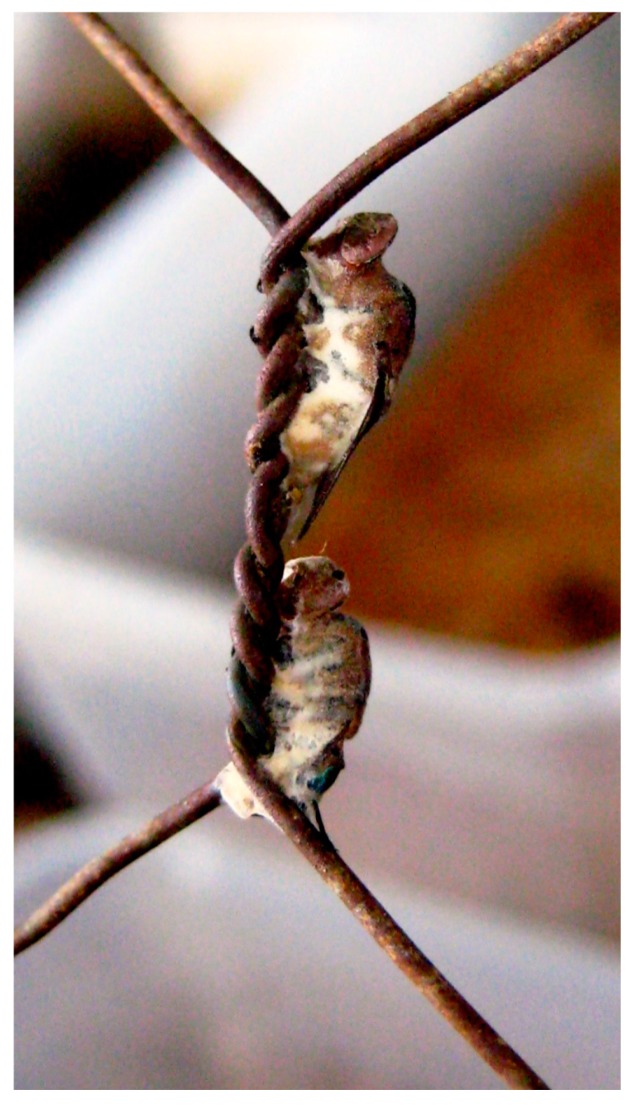
Fungus infected insects (photograph courtesy of Chin C. Heo).

Other members within the microbial consortia may have evolved phenotypes for outcompeting nearby residents. For instance, microbes may secrete antimicrobial compounds, sequester communication signals, harvest nutrients, or conduct contact-dependent killing [117]. Competition may be intense, with the outcome dependent upon several factors, including growth rate, rate of nutrient uptake, nutrient requirements, and metabolic rates. However, competition may be reduced over time through competitive exclusion, or niche partitioning via resource or spatial separation, leading to communities with a reduced local diversity of strains and species that can, nevertheless, coexist stably. These types of interactions have been described for *Lucilia sericata* (Meigen) (Diptera: Calliphoridae) and *Proteus mirabilis* I [43,118,119]. With this system, *P. mirabilis* resides within the gut of immature *L. sericata*. The bacteria release miribilicides, which potentially protect the developing larvae from pathogenic bacteria [118]. However, in some cases, microbes, such as fungi, could instead hijack the insect and use it as a dispersal mechanism [120] (Figure 3).

**Figure 4:**
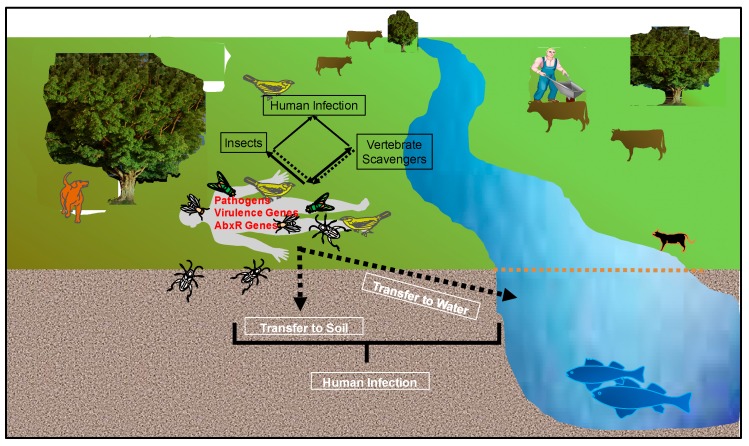
Potential mechanisms of pathogen and microbial gene dispersal following host death. Pathogens and genes can be exchanged and dispersed through scavengers, insects, soil, and water, which can lead to human transmission and infection. Double arrows are used to describe the transfer of microbes and genes between human remains and insects and scavengers. AbxR: Antibiotic Resistant. Figure designed by Heather R. Jordan and Jeffery K. Tomberlin.

Though pathogens may only represent a very small portion of all microorganisms, the dispersal and proliferation of these organisms from a cadaver is of concern for public health. The commensal microbial community associated with the living host includes opportunistic pathogenic microorganisms that are held to a particular realized niche in the healthy host, and cause disease during an immunocompromised host state. Following host death, immune function ceases over the first several hours [94] creating an optimal environment for these pathogens to proliferate. Additionally, the antemortem health state or manner of death of the individual may also allow for strict or obligate pathogen proliferation. Depending upon cadaver placement, these organisms may be dispersed by vertebrate scavengers, insects (previously discussed), in the soil, or in groundwater, where fecal coliforms have been found to proliferate and remain viable for extended periods of time [121]. Zoonotic bacterial and fungal pathogens that typically spend their lifecycle in domestic animals without causing disease in their typical hosts can also survive and, under favorable conditions, reproduce in terrestrial and aquatic environments, and can lead to severe or lethal disease when transmitted to people [122,123]. Finally, dispersal and transmission of antibiotic resistance and virulence genes among microorganisms, including pathogens, from these cadavers is an ever-present and alarming threat (Figure 4).

For many pathogens, a specific host may be necessary for survival, so, following host death, these organisms may die out without successful transmission. Other microbes, however, have adapted survival strategies in response to adverse environmental conditions, battling for survival, facing limited nutrient availability, osmotic stress, large variations in temperature and pH, and predation [124,125,126]. For example, *P. mirabilis* survives in blow fly larvae [43].

Some strategies include formation of endospores, entry into the viable, but non-culturable, state, or survival in protists [127]. These bacterial pathogens, such as (though certainly not limited to) pathogenic strains of *E. coli*, *Salmonella*, or *Clostridium* ssp. can also proliferate outside animal hosts. Many of these, such as with pathogenic strains of *E. coli*, have been found to die quickly in fresh water (one day), sediments (1.5 days), or soil (three days) [126,128,129,130]. Furthermore, in microcosms, host-adapted *E. coli* could not survive without the addition of readily usable carbon sources, or removal of competitor microflora, suggesting that host adapted *E. coli* are unable to acquire and compete for nutrients under starvation conditions [126,131]. In contrast, other host-adapted pathogens, including nearly all fungal species, can survive for long periods of time outside of host environments in soil, water, and sediments [122,125,132,133,134,135].

Bacterial and some fungal pathogen dispersal and proliferation in terrestrial and aquatic environments is associated with attachment to particles. This attachment is influenced by various factors, including temperature, microbial genotype, soil particle size, organic matter, pH, ionic strength, dissolved nutrients, and turbidity [136,137,138]. For instance, surface charges on clay particles retards microbial diffusion by slowing the water infiltration, but also allows for increased survival with increased clay content and adsorption [139], but sloped surfaces increase the risk of dispersal [140,141]. Additionally, high soil pH is also favorable to microbial transport where twice as many bacteria were retained in the soil matrix of a microcosm at a low pH [142]. In that system, soil type and bacterial size also influenced pathogen movement [142]. Particles in aquatic systems are also often associated with nutrients and particulate carbon and phosphorus, in particular, both of which can be limiting substrates for bacterial growth and, in turn, can affect pathogen survival in non-host, aquatic environments [143]. Fungi also have an additional array of dispersal mechanisms. Dispersal of many pathogenic fungi is dependent on birds, insects, and other animals, as well as wind and water. Fungal spores can also be actively discharged into the air or in water through attachment to a leaf or other surface, and can travel for long distances [144]. Furthermore, some non-motile fungi can be dispersed by bacteria [145].

Flies and other insects are also important for pathogen dissemination [146] as previously mentioned. Furthermore, dispersal could also potentially be linked with insects serving as vectors [147,148,149]. For instance, a number of fungi of medical importance were isolated from cockroaches in hospital wards and residential areas [150]. Pathogenic *Salmonella* can survive in house flies for up to a month [151], and in infected blowfly pupae for up to 29 days [152]. Additionally, flies can transmit *Salmonella* in their excreta [149,153]. Many pest species, such as flies and cockroaches, have also been known to harbor or transfer pathogens, such as *Enterobacteriaceae, E. coli*, *Staphylococcus, Campylobacter*, *Claviceps purpurea*, *Candida* ssp., and others [150,154,155,156].

The common factor in the ecology of several species of these insects is their utilization of decomposing organic materials as food sources for the adults and developmental media for their maggots (larvae). For instance, flies, cockroaches, and other insects are attracted to VOCs produced within the carrion resource [157,158]. Considering that these resources are all potentially associated with pathogens, and many instances associated with human activity, the potential for these pest species becoming contaminated can be quite high. 

However, as previously stated, the majority of non-host environments are characterized by thermal variability, high osmolarity, pH fluctuations, and low nutrient availability, suggesting that a stress response is activated among successful pathogens. Therefore, having an understanding of the spatial distribution and temporal variability of the microbial pathogens responsible for these diseases is essential. Furthermore, specific pathogen characteristics should be assessed, along with corresponding environmental parameters in order to determine successful proliferation, dispersal, and transmission as understanding the factors that control their distribution from a carrion resource is a prerequisite for reducing the human health risks. 

## 4. Conclusions

Decomposing remains are a resource for many organisms including, but not limited to, microbes and insects. Biodiversity and succession of each of these during vertebrate decomposition is dependent upon interactions between the two, as well as discrete abiotic and biotic factors specific for each kingdom. The goal of this publication was to provide a general overview of the importance of microbes, in particular bacteria and fungi, to the decomposition process and convince the reader that, while they are not visible, they aid in regulation of arthropod behavior as related to forensic entomology. Furthermore, it was our intent to discuss instances that where microbial physiology and insect behavior transect, inter-kingdom communication would likely occur, and the potential consequences of such interactions in relation to decomposing remains. A discussion of the influence of many abiotic and biotic factors impacting microbial activity in association with decomposing remains has been presented; however, this list is not comprehensive by any means, as the space required to discuss everything is beyond its scope, and we hope this work inspires others of like kind. However, while the goal of this publication was to provide an overview, a secondary goal was to bring awareness to forensic microbiology, demonstrate its relevance to forensic entomology, and potentially inspire future research on the topic to better explain the time of death as related to arthropod attraction, colonization, and utilization of vertebrate remains, with the ultimate goal of the integration of multidisciplinary investigations for obtaining useful forensic evidence.

## Figures and Tables

**Table 1:** Temperature changes leading to cellular changes in microbial cells, and the resulting cellular outcomes.

Temperature Condition	Mechanism	Effect	Citation
Cold shock	Membrane rigidity	Decreased energy, decreased fluidity	[52]
Cold shock	Ice crystals form (in presence of water)	Cell lysis	[54]
Cold shock	Enzyme activity slows or ceases	Cell Growth slows or ceases	[54]
Optimal temperature	Increased membrane fluidity	Cell Growth increases	[55]
Optimal temperature	Increased metabollic activity and enzyme rate	Cell Growtn increases	[55]
Heat Shock	Protein denaturation	Cell Growth ceases	[56]
Heat or cold shock	Regulation of protein folding	Regulation of protein secretion	[57]
Extreme cold or heat shock	Increased membrane permeability and potential	Effects on active transport and ATP synthesis; Inability to form functional, stable organelles (eukaryotes)	[52,53,58]
Extreme cold or heat shock	Increased pH	Effects on DNA transcription, protein synthesis, and enzymatic activity	[58,59]
Extreme cold or heat shock	Increased reactive oxygen species	Cell death	[58]
